# Influence of Scribes on Patient-Physician Communication in Primary Care Encounters: Mixed Methods Study

**DOI:** 10.2196/14797

**Published:** 2019-07-11

**Authors:** Shivang U Danak, Timothy C Guetterman, Melissa A Plegue, Heather L Holmstrom, Reema Kadri, Alexander Duthler, Anne Yoo, Lorraine R Buis

**Affiliations:** 1 Department of Family Medicine University of Michigan Ann Arbor, MI United States; 2 Department of Interdisciplinary Studies Creighton University Omaha, NE United States; 3 Department of Family Medicine University of Colorado School of Medicine Aurora, CO United States; 4 College of Pharmacy University of Michigan Ann Arbor, MI United States

**Keywords:** electronic health records, documentation, medical informatics

## Abstract

**Background:**

With the increasing adoption of electronic health record (EHR) systems, documentation-related burdens have been increasing for health care providers. Recent estimates indicate that primary care providers spend about one-half of their workdays interacting with the EHR, of which about half is focused on clerical tasks. To reduce documentation burdens associated with the EHR, health care systems and physician practices are increasingly implementing medical scribes to assist providers with real-time documentation. Scribes are typically unlicensed paraprofessionals who assist health care providers by documenting notes electronically under the direction of a licensed practitioner or physician in real time. Despite the promise of scribes, few studies have investigated their effect on clinical encounters, particularly with regard to patient-provider communication.

**Objective:**

The purpose of this quasi-experimental pilot study was to understand how scribes affect patient-physician communication in primary care clinical encounters.

**Methods:**

We employed a convergent mixed methods design and included a sample of three physician-scribe pairs and 34 patients. Patients’ clinical encounters were randomly assigned to a scribe or nonscribe group. We conducted patient surveys focused on perceptions of patient-provider communication and satisfaction with encounters, video recorded clinical encounters, and conducted physician interviews about their experiences with scribes.

**Results:**

Overall, the survey results revealed that patients across both arms reported very high satisfaction of communication with their physician, their physician’s use of the EHR, and their care, with very little variability. Video recording analysis supported patient survey data by demonstrating high measures of communication among physicians in both scribed and nonscribed encounters. Furthermore, video recordings revealed that the presence of scribes had very little effect on the clinical encounter.

**Conclusions:**

From the patient’s perspective, scribes are an acceptable addition to clinical encounters. Although they do not have much impact on patients’ perceptions of satisfaction and their impact on the clinical encounter itself was minimal, their potential to reduce documentation-related burden on physicians is valuable. Physicians noted important issues related to scribes, including important considerations for implementing scribe programs, the role of scribes in patient interactions, how physicians work with scribes, characteristics of good scribes, and the role of scribes in physician workflow.

## Introduction

Recent estimates suggest that primary care physicians spend about one-half of their workday, nearly 6 hours, interacting with the electronic health record (EHR) during and after clinic hours [1]. Nearly one-half of this time (157 minutes, 44.2%) is spent on clerical tasks, and an additional 85 minutes (23.7%) is spent on managing inboxes [1]. This has led to providers spending more time on clerical duties than with patients, which may have significant consequences.

EHRs have been widely adopted in the United States, which is largely driven by the Centers for Medicare & Medicaid Services (CMS) EHR Incentive Program, where over 95% of CMS eligible and critical access hospitals [2] and over 60% of office-based physicians [3] have met the Stage 1 Meaningful Use criteria. Although EHRs can positively affect patient safety, continuity of care, and compliance with regulatory and billing requirements [4], EHR implementation is still associated with negative outcomes, with some evidence suggesting that documentation time increases as a result of EHR implementation [5]; however, it is not clear whether this effect persists over time. Moreover, one recent study conducted in ophthalmology suggests that over the course of a decade, EHR documentation time and note length increased significantly [6].

In an effort to reduce documentation burdens, health care systems and physician practices are increasingly implementing medical scribes. Scribes are typically unlicensed paraprofessionals who assist health care providers by documenting notes under the direction of a licensed practitioner or physician in real time [7], although nurses or medical assistants (MAs) may also serve as a scribe. Scribes have been shown to reduce physician charting time and improve work-life balance, all while having good patient acceptance [8]. Moreover, scribes may yield a positive return on investment and may help generate revenue [8].

Despite the promise of scribes, few studies have investigated their effect on clinical encounters, particularly regarding patient-provider communication. This study aimed to understand how patient-physician communication differed with and without the use of scribes.

## Methods

### Overview

We employed a convergent mixed methods design for this quasi-experimental pilot study. We also included a sample of patient encounters that were randomly assigned to a scribe (scribe present) or nonscribe (no scribe) group. To determine the effect of scribes, we collected patient surveys on perceptions of patient-provider communication and satisfaction with the encounter, video recordings of clinical encounters, and physician interviews focused on scribes. All methods were approved by the University of Michigan IRBMED Institutional Review Board (HUM00123396).

### Recruitment

#### Physician/Scribe Recruitment

Family medicine physicians from a large Midwestern academic medical center known to be using scribes were recruited via targeted emails. Participating physicians consented to allow clinical encounters to be videotaped and to complete an audio-taped interview at the conclusion of the study. Once the physicians provided consent, we recruited their scribes via targeted emails. To incentivize participation, physicians and scribes received US $100 and US $25, respectively.

#### Patient Recruitment

We reviewed clinic schedules for enrolled physician-scribe pairs to identify potentially eligible participants. We excluded new patients or those scheduled for a health maintenance exam, as these encounters typically include full physical exams, where the expectation of having the patient disrobe would be most common, and may be considered too sensitive to record by some patients. Potential participants were contacted by phone 2 days prior or approached in the clinic waiting room before their scheduled appointment. To be eligible, patients were required to consent to having their encounter videotaped and complete a survey after their encounter. Patients were awarded US $25 for their participation after survey completion.

### Study Procedures

#### Video Recording of Encounters

GoPro Hero4 Session video cameras (model number: HWRP1; GoPro, Inc, San Mateo, CA) were set up; removed by the study staff immediately before and after the encounter; and turned on by study staff, scribes, or physicians immediately after the physician entered the room. Physicians were instructed that they, or the scribe, could turn off the camera at any time for any reason.

#### Postencounter Patient Survey

After the encounter, patients completed a survey assessing demographics; experience with the care team; and perceptions of satisfaction with the care team, the encounter, and role of the EHR. The survey also included the Communication Assessment Tool (CAT), a 15-item instrument written at a fourth-grade reading level and using a 5-point Likert-type response scale, to measure patients’ perceptions of physician performance regarding interpersonal and communication skills [9,10].

#### Physician Interview

After patient data collection was complete, physicians completed semistructured interviews focused on experience and workflow with scribes, communication during encounters, and additional suggestions they had for future scribe usage (Multimedia Appendix 1).

#### Retrospective Chart Review

To identify whether scribes had any impact on encounter timing, we performed a retrospective chart review for all included encounters. We conducted the chart review to identify scheduled appointment time, recorded time vitals, and calculated the time to chart close.

### Analysis

#### Video Recording

Three researchers coded video recordings. Each video was coded by two people to ensure accurate analysis. Codes between the two coders were compared, and the third coder resolved disputes. Videos were coded using the Interview Assessment Tool (IAT), which is a rubric used to assess physician communication skills with patients and has been used to teach and educate medical students on effective communication [11]. The IAT comprises 13 domains, scored from 1 (worst) to 4 (best). An overall IAT score was computed across the 13 domains, with a maximum score of 52 points. Domains included in the IAT were Introductions, Patient Eye Contact, Nonverbal Communication Cues, Listening, Questions, Wait Time, Interest/Concern, Organization, Information Gathering, Focus, Empathy, Awareness of Unspoken Issues, and Closure of the Encounter.

Coders also assessed physician/scribe introductions, the percentage of time spent looking at the computer (in increments of 5%; based on approximate time on computer divided by total encounter time), uses of the computer (eg, notes, looking up lab results); patient-physician interaction, patient-scribe interaction, interruptions, space (congestion, emptiness, and layout of room), and medication order/entry.

Independent sample *t* tests compared the average appointment duration, percent of time spent looking at the computer, number of problems addressed, and number of orders placed between scribed and nonscribed encounters. IAT items were not statistically compared between groups due to the heavy skew and lack of variability in item values across encounters. Medication orders were assessed descriptively and checked for errors by comparing the EHR to the notes taken when recording.

#### Patient Survey

Patient demographics were compared between groups using Chi-square and *t* tests. The CAT was scored by taking a mean response of the item and computing the proportion of “excellent” responses for each respondent [9]. The values were then compared between groups using *t* tests. Additional items on the survey were compared using Chi-square or *t* tests between groups when permitted by data variability.

#### Physician Interview

We conducted thematic text analysis, consisting of three major tasks: reading through data, assigning codes to relevant text segments, and identifying major themes across codes [12]. Two individuals coded the data initially and discussed codes to develop consensus and refine the codebook. Coders next applied codes across interviews and open-ended comments in the video assessment tool. Looking for patterns and commonalities, codes were grouped into major themes. Finally, we integrated the qualitative themes about the process of using scribes from physicians’ perspectives by examining each theme in comparison to the key quantitative results.

#### Retrospective Chart Review

From the extracted chart data, we assessed time to close charts for each encounter, calculated as time from the scheduled appointment time to chart closure, as well as time from vital sign recording to chart closure. Linear regression models were performed for both outcomes, with time to chart close as the outcome, whether an encounter included a scribe as a primary predictor of interest, and an additional covariate for provider.

## Results

### Participants

We recruited three physician-scribe pairs and 34 patients (19 and 15 randomized to scribed and nonscribed encounters, respectively). Only 31 recordings were obtained due to technical and user error, resulting in 17 scribed and 14 nonscribed recordings. Participants were predominantly of white race (79.4%) and male gender (67.7%), with income >US $50,000 (73.5%). Participants were, on an average, 51.1 years (SD 19.1) of age and equally divided between having a bachelor’s degree or higher education and some college or less education (Table 1).

### Effect of Scribes on Patient-Physician Communication and Satisfaction

Patients reported very high satisfaction with physician communication. Communication scores from the CAT were positively skewed, with no respondents rating anything less than “Good,” and the majority of items rated “Very Good” or ”Excellent.” Overall, 100% of respondents reported that their physicians’ communication was Excellent/Very Good in terms of greeting patients in a way that made them feel comfortable, treating patients with respect, showing interest in patients’ ideas about their health, understanding main health concerns, paying attention to the patient, giving as much information as the patient wanted, talking in terms that the patient could understand, checking to be sure the patient understood everything, discussing next steps, and showing care and concern. Neither the mean score of the CAT nor proportion of excellent responses had much variability; 21 of the 34 patients responded “Excellent” to all 14 questions (Table 2). No significant differences were found in communication scores between the scribed and nonscribed encounters.

**Table 1:** Patient demographics.

Demographic			Overall (N=34)		Scribed encounters (n=19)		Nonscribed encounters (n=15)		*P* value^a^
Age (years), mean (SD)			51.1 (19)		52.8 (5)		49 (4)		0.58
**Gender, n (%)**									**>.99**
	Male	23 (68)		10 (67)		13 (68)			
	Female	11 (32)		5 (33)		6 (32)			
**Race, n (%)**									**.43**
	White	27 (79)		14 (74)		13 (87)			
	Other	7 (21)		5 (26)		2 (13)			
**Education, n (%)**									**>.99**
	Less than bachelor’s degree^b^	17 (50)		9 (47)		8 (53)			
	Bachelor’s degree or higher	17 (50)		7 (47)		10 (53)			
**Income (US $), n (%)**									**.70**
	<50,000	8 (24)		5 (28)		3 (20)			
	≥50,000	25 (74)		13 (72)		12 (80)			
	Unknown	1 (3)		N/A^c^		N/A			

^a^Independent samples *t* test for age and the Fisher exact test for all other variables.

^b^Recoded any “other” responses to less than bachelor’s degree, as most were “some college.”

^c^N/A: not applicable.

**Table 2:** Communication Assessment Tool score.

Parameters	Overall (N=34)	Scribed encounters (n=19)	Nonscribed encounters (n=15)	*P* value
Score, mean (SD)	4.9 (0.27)	4.84 (0.29)	4.86 (0.27)	.84
% excellent responses, mean (SD)	86.3 (24.7)	85.3 (26.6)	87.6 (23.0)	.79

All patients had positive assessments of their interaction with their physicians, their physician’s EHR use, and satisfaction with care. No significant differences were found between groups in terms of these aspects; however, response variability was low among both groups, with most items skewed positively (Table 3). The exception was only 52.6% of patients in the scribed encounters compared to 93% in the nonscribed encounters who indicated that physicians used the computer (*P*=.02).

The above mentioned findings were supported by video analysis, where both groups scored high on 12 of the 13 IAT domains, with little to no variability. The Introduction domain was discarded, as most recordings (n=23) started after physicians entered the room and presumably made their introductions. For the remaining 12 domains, three domains (Questions, Wait Time, and Concern) had no variability between groups, with all recordings coded as having the highest performance possible. An additional eight domains (Eye Contact with Patient, Nonverbal Communication, Listening, Organization, Information Gathering, Focus, Empathy, and Awareness of Unspoken Issues) had two or fewer videos in either group coded as 3. No encounter was coded as having the poorest performance (score of 1 or 2) on any domain. This suggests that physicians consistently demonstrated high performance across all IAT domains. The Closure domain had the largest difference between groups, with 50% of nonscribed encounters showing a score of 3 and 50% showing a score of 4; in addition, 18% of scribed encounters showed a score of 3 and 82% showed a score of 4. The presence of scribes was not associated with performing less (≤3 on IAT) than the highest category of performance (4 on IAT).

### Effect of Scribes on Clinical Encounter

Video recordings revealed that scribes had little effect on encounters. Although the scribed encounters were slightly shorter, this difference was not significant (mean 15.6 [SD 5.4] min vs mean 16.5 (SD 6.7) min; *P*=.70). When scribes were present, physicians spent slightly less time looking at the computer (mean 16.1% [SD 15.5%]) than when scribes were absent (mean 29.8% [SD 23.7%]; *P*=.06). Neither the number of problems addressed nor the number of orders placed differed significantly between groups. Across all visits, the mean number of problems addressed was 3.4 (SD 1.4; scribe: mean 3.3 [SD 1.4] vs nonscribe: 3.6 [SD 1.6]; *P*=.60) and the mean number of orders placed was 0.9 (SD 1.0; scribe: mean 0.8 [SD 0.8] vs nonscribe: mean 1.1 (SD 1.2); *P*=.41). Linear regression results revealed that there were no significant differences between the scribed and nonscribed encounters with regard to the time to close charts.

**Table 3:** Patient survey data responses categorized by scribe group.

Item		Patient responses, n (%)				
		Strongly agree	Somewhat agree	Neutral	Somewhat disagree	Strongly disagree
**The doctor paid attention to me throughout the entire clinic visit.**						
	Scribe (n=19)	18 (94.7)	1 (5.3)	N/A^a^	N/A	N/A
	Nonscribe (n=15)	14 (93.3)	1 (5.3)	N/A	N/A	N/A
**My interactions with my doctor was disrupted by the computer system.**						
	Scribe (n=18)	2 (11.1)	1 (5.6)	N/A	1 (5.6)	14 (77.8)
	Nonscribe (n=15)	N/A	N/A	1 (6.7)	3 (20.0)	11 (73.3)
**The usage of the computer system has made my medical care better.**						
	Scribe (n=18)	6 (33.3)	7 (38.9)	5 (27.8)	N/A	N/A
	Nonscribe (n=15)	7 (46.7)	5 (33.3)	2 (13.3)	1 (6.7)	N/A
**The usage of the computer system has made my medical care safer.**						
	Scribe (n=19)	7 (36.8)	4 (21.1)	7 (36.8)	1 (5.3)	N/A
	Nonscribe (n=15)	6 (40.0)	3 (20.0)	6 (40.0)	N/A	N/A
**I am comfortable with someone other than my physician taking notes.**						
	Scribe (n=19)	14 (73.7)	5 (26.3)	N/A	N/A	N/A
	Nonscribe (n=15)	8 (53.3)	5 (33.3)	2 (13.3)	N/A	N/A
**All of the reasons I came to see the doctor were addressed today.**						
	Scribe (n=19)	18 (94.7)	1 (5.3)	N/A	N/A	N/A
	Nonscribe (n=15)	14 (93.3)	1 (6.7)	N/A	N/A	N/A
**I was satisfied with my care today.**						
	Scribe (n=19)	16 (84.2)	3 (15.8)	N/A	N/A	N/A
	Nonscribe (n=15)	13 (86.7)	2 (13.3)	N/A	N/A	N/A
**I felt that there were too many people in the room.**						
	Scribe (n=19)	N/A	1 (5.3)	1 (5.3)	2 (10.5)	15 (79.0)
	Nonscribe (n=15)	N/A	N/A	5 (33.3)	N/A	10 (66.7)

^a^N/A: not applicable.

Although it was standard protocol at this institution for medication orders to be entered by physicians or pended (but not signed) by medical assistants, scribes were restricted by institutional policy from pending medication orders. However, during the time of our data collection, the family medicine clinics where data collection occurred were participating in an institutional pilot that granted permission for scribes to pend (but not sign) medication orders during an encounter. There were 27 medication orders (10 renewals) across 18 encounters. Most medication orders were entered by the physician, and only nine were entered by the scribe, all of which were new orders. No medication errors were identified when comparing video recordings to EHR data.

### Physician Perceptions of Scribes

Qualitative interviews yielded five major themes regarding scribes (also see Multimedia Appendix 2).

#### Theme 1: Considerations for Implementing Scribe Programs

Physicians noted considerations such as the level of scribe training, scribe understanding of privacy, and the preparation of providers (eg, EHR proficiency) before implementing scribes. Physicians noted benefits of consistency among scribes and consequences of turnover. One physician mentioned the possibility of additional scribe tasks in a combined role with MAs, thereby reducing turnover.

#### Theme 2: Role of Scribes in Patient Interactions

Physicians discussed their views on the role of scribes with patients. One physician expressed that scribes should have minimal interaction with patients after a brief greeting. When asked about scribe gender, physicians consistently reported no gender-related effect, but some noted that they often have scribes leave the room during sensitive physical exams.

#### Theme 3: How Physicians Work With Scribes

Providers discussed strategies for work with scribes to improve documentation quality, workflow efficiency, and response to health maintenance prompts. When working with a scribe, physicians recommended placing them behind or off to the side to allow the provider to focus on the patient.

#### Theme 4: Characteristics of a Good Scribe

Physicians identified several characteristics of good scribes, such as the ability to adapt and make changes, remain quiet, learn terminology, use the EHR, and employ basic social and communication skills. Additional noted qualities were focus, investment in the job, and a professional demeanor.

#### Theme 5: The Role of Scribes in Physician Workflow

Physicians indicated the need to consider the role of scribes in all phases, from visit preparation to introduction, assessment, documentation during the visit, and summary of the plan in the record.

## Discussion

### Principal Findings

Our results showed that scribes did not have any significant impact on measures of patient satisfaction or the encounter itself, suggesting that from a patient’s perspective, scribes are acceptable to patients.

Overall, patients were pleased with the medical care they received and were satisfied, regardless of scribe presence, which is consistent with the literature [13,14]. Although, Pozdnyakova et al also found no differences in patient satisfaction between patients in scribed and nonscribed encounters, they found that compared to patients aged ≥65 years, younger patients were more likely to find physicians attentive and provide more education when scribes were present [15]. Although our sample was small and had too little variability in patient satisfaction measures to find differences between older and younger patients, our findings merit further investigation.

Our study also showed that scribes had little impact on the encounter itself. When scribes were present, physicians spent more time in the encounter looking at the patient as opposed to the computer, a finding that was marginally significant. This is consistent with other similar findings in the literature [16]. Patients noticed that that their physician was the professional using the computer more frequently in nonscribed encounters as compared to scribed encounters (93% vs 52.6%), but this factor too had little impact on patients’ perceptions of the encounter. Although scribed encounters were, on an average, about 1 min shorter, this marginal efficiency was not statistically significant, which is contrary to other studies that found efficiencies in terms of physicians’ ability to see more patients per hour [16]. It is possible that our lack of a significant difference is due to our small sample size. Regardless, our findings support the idea that scribes do not lengthen clinical encounters and may even save time.

Our findings that scribes do not affect patient satisfaction and that they have little effect on the encounter itself are important. Physician participants reported positive experiences and satisfaction with their scribes, which has been reported elsewhere [8,14,17,18]. Administrative duties require substantial physician time and have affected physicians’ perceptions of ability to deliver high-quality care, career satisfaction, and burnout [19]. In fact, the demands of documentation and EHR use are a chief contributor to physician burnout [20,21]. In a large national study by Shanafelt et al, physicians’ satisfaction with EHRs and computerized physician order entry was generally low, and physicians who used these systems were at higher risk for professional burnout [22]. The topic of physician burnout has been gaining national attention, as it has been strongly correlated with health issues such as depression, drinking problems, and cardiovascular and digestive disorders as well as use of sedatives and overeating [23]. Previous research has shown that providers find scribes valuable and that they reduce documentation time [17]. Future work should seek to more clearly elucidate the relationship between scribes and physician burnout.

### Interpretation of the Integrated Results

Although our quantitative results suggest support for scribes in clinical encounters, integrating results from our physician interviews highlight additional important context. The negative effect of scribe turnover was highlighted by nearly every physician. Scribe vendor services often employ young professionals in their gap year before medical school [24]. This creates a system where a cadre of scribes enters the workforce for 9-12 months before leaving for medical school, which creates a lack of consistency within the clinic and has significant transaction costs.

This discussion coincides with one of our providers theorizing about expanding the role of a scribe, allowing them to perform duties of an MA, or vice versa, where an MA can also assist in documentation. It is important to note that although we focused our research in a health system that relies on vendor scribes, other models have been reported in the literature, such as using nurses or MAs as scribes in addition to their regular duties [15,25]. The medical background and training for this cross-over type of role would need further investigation. Finally, although scribes cost money, they are often cost neutral [8]. McCormick et al. found that the return-to-investment ratio was greater than 6:1 when using scribes but had no effect on patient satisfaction [18].

### Limitations

An important limitation of our study was the small sample of physician-scribe pairs. With only three physicians enrolled, we were limited to their patients and only their insight, which limits generalizability. Moreover, we did not collect data from scribes. Future work should incorporate scribes as research participants to obtain their perspective on workflow and identify challenges in working with different physicians and specialties. Another limitation of our approach is the fact that physicians and scribes were completely aware of which encounters were being recorded (in most cases, it was the physician who started the recording). Because of this knowledge, it is possible that physicians and scribes may have altered their behavior within encounters. The only way to mitigate this possibility would be through discreet recording, which is not ethical or appropriate. It is possible that with more recorded encounters, any potential effects of recording may dissipate; however, this is not guaranteed. We must also note that despite the fact that survey responses were not shared with physicians, patient participant survey responses were not anonymized to study staff. Because responses were not completely anonymous to study staff, it is possible that patient participants may not have answered survey items truthfully, which may have contributed to the lack of variability and positive skew in survey results. Although scribes are often employed to help reduce documentation burdens among physicians, we did not design this study to verify whether that was the case. Despite finding a nonsignificant difference in encounter duration, with scribed encounters lasting for a slightly shorter duration than nonscribed encounters, we do not know if the presence of a scribe had an impact on documentation burden. Future work should investigate whether these theoretical efficiencies are actually established both inside and outside clinical encounters. Finally, we did not assess physician EHR literacy or comfort, which may have a possible effect on individuals’ use of scribes. Future work should seek to better understand the relationship between physician EHR literacy and scribe-related efficiencies.

## Appendix

Multimedia Appendix 1 Physician interview guide.

Multimedia Appendix 2 Themes, related codes, and illustrative quotes from physician interviews concerning their use of scribes.

## Abbreviations

CAT: communication assessment tool
EHR: electronic health record
IAT: interview assessment tool
MA: medical assistant
