# Supplementary material for: Influence of Scribes on Patient-Physician Communication in Primary Care Encounters: Mixed Methods Study
Source: JMIR Med Inform. 2019 Jul 11;7(3):e14797. doi: 10.2196/14797 (PMC6657447; doi:10.2196/14797)
Supplement: Multimedia Appendix 1 [file medinform_v7i3e14797_app1.pdf]

Multimedia Appendix 1. Physician interview guide.

[NOTE: THIS DOCUMENT IS TO BE TREATED AS A GUIDE, NOT A SCRIPT. THE INTERVIEWER MAY DEVIATE FROM THE WRITTEN TEXT TO CLARIFY RESPONSES, FURTHER EXPLORE A TOPIC, AND TO ENSURE ADEQUATE INFORMATION IS OBTAINED]

Experience:

First, I have some broad questions about your experience with scribes.

1. What has your experience with scribes been like so far?

2. What characteristics of scribes are most effective?

3. What characteristics of scribes are least effective?

4. How does the gender of the scribe play a role in the encounter?

    a. Do you have scribes present during all or certain physical examinations?

5. What are your thoughts on the training and education of the scribes?

6. How has your experience been in terms of professionalism with scribes?

7. If you were able to expand the services the scribe can do for you, what would you request?

8. How has the mandate of having EHR affected your communication with patients?

    a. How does the use of scribes play a role in the EHR?

Workflow:

Now, we are going to change focus a little and talk about scribes and your workflow specifically.

9. Please walk through a typical encounter from start to finish.

10. How are scribes involved before going into the room?

    a. How are scribes involved in the room?

    b. How are they involved after the visit?

c. How do you review documentation the scribes have entered? [in the clinical encounter itself]

11. How important is it that you work with the scribe regularly?

12. How would you compare the documentation quality when working with scribes versus not working with a scribe?

<u>Communication:</u>

I want to shift one final time for the last part of our interview and ask about how you talk with patients when your scribe is present.

13. How do you interact with patients when a scribe is present?

    a. How does the encounter differ from when no scribe is present?

    b. How does the patient interact with the scribe?

    c. How do you maintain patient privacy?

14. How do you talk with patients when a scribe is present?

a. How do you introduce the scribe?

15. Tell me about physical layout and how you move around the room (i.e. where you sit, the patient sits, where the scribe sits) when a scribe is present *(physical space, movement, set up)*

Wrap up:

16. If you were giving recommendations to a new clinic that wanted to use scribes, what would you suggest to them?

17. I appreciate your answer to all of our questions. What else might be important for us to know that we have not asked about?

Thank you very much for your time.
