# Supplementary material for: Influence of Scribes on Patient-Physician Communication in Primary Care Encounters: Mixed Methods Study
Source: JMIR Med Inform. 2019 Jul 11;7(3):e14797. doi: 10.2196/14797 (PMC6657447; doi:10.2196/14797)
Supplement: Multimedia Appendix 2 [file medinform_v7i3e14797_app2.pdf]

Multimedia Appendix 2. Themes, related codes, and illustrative quotes from physician interviews concerning their use of scribes.

Code	Description	Sample excerpts
Considerations for Implementing a Scribe Program in a Clinic		
Training Quality	Related to level of training of scribes by the vendor.	"They do a lot of pre-training and work so when they come, when they show up in the office they have a basic level of competence…"
Consistency of Scribe	Related to having a scribe regularly during encounters, as well as related scribe turnover.	"… if you don't work with scribes regularly they don't know how you like to do things and it makes it less efficient."
Cost of Scribes	The financial costs associated with using scribes.	"I guess for that clinic that was going up and running if it were primary care I would say here is an option among others. Here are the costs. Here are the risks."
Patient Privacy/Confidentiality	In relation to the privacy and confidentiality of the patient when using scribes.	"I would hope they sign a confidentiality agreement and HIPAA. I assume that they do."
Expanded Role of Scribes	Potential responsibilities that a scribe may be able to take on in the future.	"Ideally to me, the scribe and MA role could be more combined. You'd have the scribe bring the patient back and do all the

		pre-stuff. You'd have the scribe do other active roles and you'd have two scribes, one in each room and in each room they'd be taken care of by that person doing everything but I don't know if that's realistic."
Physician Skills	Skills required by physicians.	"I think it's important for the physicians before they get a scribe to actually get facile with the electronic medical record themselves…"
The Role of Scribes in Interaction		
Scribe Patient Interaction	Interactions that take place between the patient and scribe.	"Ideally besides that initial hello, they don't at all. In fact, that's the goal is to have there be no interaction at all. Although at the end a ton of patients will always be like 'I forgot about them' or 'I didn't notice they were there' or 'thank you'."
Physician Patient Interaction	Interactions that take place between the physician and patient.	"I think that engagement with patients is helpful and not having this computer in between, sort of feeling like there's a

		barrier and especially the more we look at the person I think they kind of like that."
Communicating when Scribe Present	The impact on communication between physician and patient when a scribe is present.	"I say, 'By the way. I want to introduce you to Scribe002, our scribe. She's going to be taking notes or helping me with - - making me more efficient about the writing'."
Gender Role of Scribes	The impact of how the gender of a scribe plays a role in the clinical encounter.	"I think as long as they step out for the private exams, which they do anyway, I haven't really seen a lot of impact regarding if it's male or female."
How Providers Work with Scribes		
Documentation Quality	Related to methods to improve the quality of documentation or examples of methods currently used.	"My initial notes are probably always gonna be better. I spend more time on it but in terms of capturing the information that I'm thinking of it's never going to be the same. But in terms of getting the major highlights, I think that's a good job."
Work/Workflow Efficiency	Methods to improve the overall efficiency of workflow during clinical encounters.	"I think that scribes can be very helpful in capturing data from the experience or from the patient visit, so I can concentrate on the

		patient, and actually pay attention to them, listen, actually think clearly and not worry about having to type and multitask, which I'm sure is not as effective as concentrating on the patient, which is the more important thing."
Helping with Health Maintenance Prompts	Potential and current role of scribes in reminding physicians about health maintenance prompts.	"A lot of the preventive screenings, best practice advisories, for example if they're due for a mammogram."
Examination Room	Refers to the physical layout and space of the examination room.	"The scribe is always purposely put sort of behind me and in many situations after the first few minutes the patients will forget."
Characteristics of a Good Scribe		
Adapt and Make changes	Ability of scribe to be adaptive in learning.	"…kind of being able to think on their feet quickly and if they can't I think that's probably detrimental also."
Quiet	Quiet behavior of the scribe, as not to make their presence disruptive to the clinic encounter.	"A good scribe is quiet."

Knowledgeable About Terminology	Knowledge of medical terminology used in clinical documentation and during assessments.	"They have to have some background in medical terminology. The more they have the better off they're gonna be."
Electronic Health Record (EHR) Computer Software Knowledge	Knowledge and understanding regarding the usage of EHR computer software.	"[EHR] has a number of features that are not obvious so you have to kind of know where they are, so somebody with [EHR] training."
Social Skills	Basic social and communication skills.	"...really helpful for providers not only just looking at their background in terms of their knowledge but social, personal skills I think is important."
Focused	Focused on the task at hand.	"I'll try to oftentimes repeat what the patient says so in case the scribe isn't paying attention…"
Invested	Invested in learning and gaining medical experience in the role of a scribe.	"All of these are college kids or pre-med, pre-law, pre-physical therapy, pre-PA, whatever it is, pre-professional undergraduates so by nature they're going somewhere else and this is not a long-term job for them."

Professional	Professionalism when interacting with patients, physicians, and clinic staff.	"…but I think the ones I've had so far have always been very professional."
The Role of Scribes in Physician Workflow During Clinical Encounters		
Visit Preparation	Tasks completed by provider prior to seeing patient in clinic examination room.	"I look at the computer, see usually how many patients I have, maybe briefly look at who they are, the ages and the problems and the gender and all that stuff."
Introduction	Introduction and formalities with patient.	"But as soon as we walk into the patient visit or the room, that's often saying hi, just basic introductions and then we'll start with sort of trying to find out why they're there or maybe there are things that I requested that they come."
Assessment	Physical examination and evaluation of patient in relation to reason behind visit.	"But then starting the history and then eventually the physical and then what I generally do that might be a little bit different and I tell the scribes that I often will review the certain diagnoses that they've had and sort of an assessment plan, then have them type that out."

Documentation	Entering data into the patient record in relation to the clinic visit.	“For the notes themselves, the documentation, again I try to do some during the flow of the day.”
Plan/Conclusion	Completion of the visit, including plan, medications, and next steps for patient.	“We talked about your high blood pressure. Here’s the medicines you’re taking. Here’s the new one you’re gonna start.”
